# Rotational Compression of Cylindrical Specimen As a New Calibrating Test for Damage Criteria

**DOI:** 10.3390/ma13030740

**Published:** 2020-02-06

**Authors:** Zbigniew Pater, Janusz Tomczak, Tomasz Bulzak, Łukasz Wójcik, Patrycja Walczuk-Gągała

**Affiliations:** Department of Computer Modelling and Metal Forming Technologies, Faculty of Mechanical Engineering, Lublin University of Technology, Nadbystrzycka 36, 20-618 Lublin, Poland; j.tomczak@pollub.pl (J.T.); t.bulzak@pollub.pl (T.B.); p.walczuk@pollub.pl (P.W.-G.)

**Keywords:** damage, calibrating test, rotational compression, FEM, experiment

## Abstract

The subject-matter of the article is the ductile fracture of materials—A phenomenon occurring in numerous metal forming processes. In order to prognosticate the possibility of a fracture, damage criteria are employed. Their effectiveness, however, depends on the accurate determination of the critical values of damage. These values are obtained through calibrating tests, where the stress state has to be as similar to the actual process as possible. The currently employed calibrating tests do not enable one to determine the limit values of the damage function when the Mannesmann effect occurs. Therefore it was not possible to effectively prognosticate the material fracture in the processes of cross- and skew-rolling. A new calibrating test, based on rotational compression of a cylindrical sample, in which the fractures are caused by the Mannesmann effect, was developed at the Lublin University of Technology. This test was discussed in the article, with a particular focus on the stress and strain state in the sample. A practical use of the test was presented on the example of C45 grade steel, formed in the temperature equal 1150 °C. In the research ten material damage criteria were adopted.

## 1. Introduction

Ductile fracture of materials is one of the main factors hindering the metal forming processes. For this reason, effective methods of prognosticating the occurrence of this hindrance are intensively researched. Four basic groups of fracture models are used in this research, namely continuous damage mechanics models, porous solid mechanics models, cohesive models and phenomenological models [1,2,3,4,5,6,7,8,9,10]. Application of the last group of models does not allow one to understand the mechanism of fracture, but facilitates the prognostication. Moreover, the models are easily implemented in commercial software used in mechanics, which results in their significant popularity.

The parameters characterizing the stress state play a crucial role in phenomenological models. Especially important here are the stress state invariables *p*, *q*, *r*, defined as follows:

(1) $$p=-\sigma_{m}=-\frac{1}{3}\left(\sigma_{1}+\sigma_{2}+\sigma_{3}\right)$$

(2) $$q=\sigma_{i}=\sqrt{\frac{1}{2}\left[{\left(\sigma_{1}-\sigma_{2}\right)}^{2}+{\left(\sigma_{2}-\sigma_{3}\right)}^{2}+{\left(\sigma_{1}-\sigma_{3}\right)}^{2}\right]}$$

(3) $$r={\left[\frac{27}{2}\left(\sigma_{1}-\sigma_{m}\right)\left(\sigma_{2}-\sigma_{m}\right)\left(\sigma_{3}-\sigma_{m}\right)\right]}^{\frac{1}{3}},$$

where: *σ*_1_, *σ*_2_, *σ*_3_—principal stresses—*σ_m_*—mean stress—*σ_i_*—equivalent von Mises stress.

Oftentimes, the influence of the first two invariables on the fracture of the material is expressed by stress triaxiality *η*, defined as:

(4) $$\eta =\frac{-p}{q}=\frac{\sigma_{m}}{\sigma_{i}}.$$

The influence of the third stress state invariable is expressed by the Lode angle parameter *θ*, calculated from the following dependency:

(5) $$\theta =1-\frac{2}{\pi }\arccos\left[{\left(\frac{r}{q}\right)}^{3}\right],$$

which values are in the range −1 ≤ *θ* ≤ 1.

At the end of the 1960s, McClintock [11] and Rice and Tracey proved that increasing the stress triaxiality accelerates the material fracture. The results of research conducted by Johnson and Cook [12] showed that this parameter has a much more significant influence on fracture than temperature or strain rate. Later on [13,14] it was found that depending on the stress triaxiality another fracture mechanism may occur. Should *η* ≥ 0.33, the fracture is caused by void nucleation, growth and coalescence. For *η* ≤ 0 a loss of structural integrity as a result of shear occurs. In the case when 0 < *η* < 0.33 both of the aforementioned fracture mechanisms may ensue. It was found [15] that if *η* < −0.33 material fracture does not take place. Lately, it has been reported in numerous works [16,17,18,19,20] that the third stress state invariable has a significant influence on material fracture.

In the aforementioned phenomenological models of material fracture it is adopted that the material damage is linked to the change of energy, caused by the accumulation of plastic strain, expressed by the following damage function:

(6) $$f_{i}=\int_{0}^{\varepsilon }\Phi \left(\sigma \right)d\varepsilon ,$$

where: *f_i_*—Damage function calculated on the basis of *i*-criterion, Φ(*σ*)—Function expressing the influence of the stress state on fracture, *ε*–Effective strain.

In the last few decades, numerous damage criteria were developed [21,22,23,24,25,26,27,28,29,30], varying in the use of function Φ(*σ*). A comparison of ten selected damage criteria, listed in chronological order is presented in Table 1. The aforementioned criteria were used further in the work.

In order to prognosticate the ductile fracture of the material, it is essential to know the critical value of the damage function *C_i_*, which determines the value of the damage function *f_i_* at the moment of the occurrence of cracking. The value of *C_i_* allows one to determine the fractured index *w_i_*

(7) $$w_{i}=100\%\frac{f_{i}}{C_{i}},$$

determining the risk of material fracture as a percentage. In the case when the index value is equal 100%, a cracking occurs.

Critical values of the damage function are determined experimentally, using the so-called calibrating tests. They are most easily performed in the case of sheet forming, where the plane state of strain is assumed and forming limit diagrams are developed. In order to achieve this the Nakajima test [31], Erichsen cupping test [32] or the tension of flat samples [33] are conducted.

In the case of solid shape forming or developing general solutions tests based on compression, tension or shearing of samples are performed. For example, Wierzbicki et al. [17,34] presented a group of ten tests listed in Table 2. In this group the following tests are included: tension of smooth bars, tension of bars with small and big notches, tension of bars in the plastic plane state of strain, tension of flat grooved plates, torsion or shear, cylinder upsetting, equi-biaxial tension in the plastic plane state of strain, compression in the plastic state of strain and compression of notched bars. Upon applying the aforementioned tests to samples of varied dimensions, one is able to obtain fifteen limit values determined for various values of stress triaxiality and Lode angle parameter, which is sufficient for a complex determination of the critical values of the damage function. This methodology was used independently by Khan and Liu [35] as well as Lou and Huh [36] to determine various fracture criteria for Al 2024-T351 alloy. An alternative for the presented methodology is using the simultaneous tension and torsion of tube-shaped samples with notches [20,37,38]. According to Papasidero et al. [39], employing various combinations of tension and torsion allows for conducting cracking tests for the stress triaxiality in the range from zero (pure shear) to c.a. 0.58 and for the Lode angle parameter in the range from 0 to 1. It is also to be mentioned that the aforementioned calibrating tests are conducted in room temperature (cold forming) and the obtained critical values of the damage function cannot be used in hot forming processes. Thus, new methods of calibration, allowing one to determine such limit values of the damage function in the state of strain that would be as close to the real process as possible, are still researched.

A new method for calibrating the damage criteria, based on rotational compression of cylindrical samples, was developed at the Lublin University of Technology. The presentation of this method is the main objective of this study. The state of stress in the axial area of the sample, occurring in this test, causes the material to crack as a result of the so-called Mannesmann effect. A similar state of stress occurs in numerous industrial processes, such as cross-wedge rolling, helical rolling and punching according to the Mannesmann method [41,42,43,44]. For this reason, the limit values of the damage function obtained using the new test have a significant utilitarian meaning. 

## 2. Material and Methods

**Figure 1:**
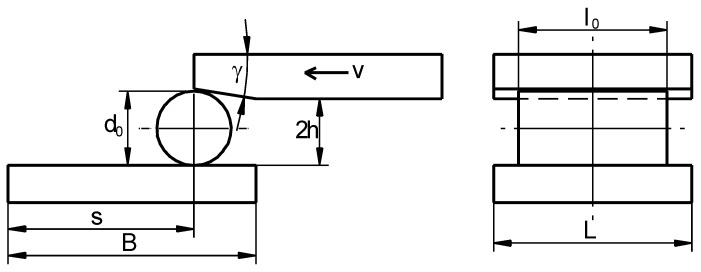
Scheme of the rotational compression test of bars, performed using two flat tools.

The new rotational compression test (Figure 1) comprises of forming a cylindrical sample with the diameter *d*_0_ using two flat tools, with their working zones situated at the distance 2*h* smaller than *d*_0_. The upper tool, moving in the plane motion with the speed equal *v* in the input zone has an undercut/notch enabling one to put the sample in the working zone of the tools. During the rotational compression in the vertical direction, the sample is put in motion by the friction forces and rolled on the path *s* on the unmoveable nether tool. Applying such load to the sample causes a variable state of compressive-tensile stress to occur in the axial area of the sample, which causes the material to crack (Mannesmann effect). The occurrence of fracture depends on the following parameters: ratio of the *l*_0_/*d*_0_ dimensions of the sample, length of the forming path s and the reduction ratio *δ* defined as:

(8) $$\delta =\frac{d_{0}}{2h}.$$

In order to determine the critical value of the damage function in the rotational compression test, one ought to experimentally determine (with *l*_0_/*d*_0_ and *δ* given) the length of the *s* path, at which the material cracking occurs. Further on, with the same parameters, the value of the damage function is to be numerically modeled and determined (at the time and in the place of the cracking), which will be the searched value.

Further in the study, an example of the usage of the rotational compression test was applied in order to determine the critical value of the damage function *C_i_* for C45 grade hot-formed steel is presented. In the test samples of the diameter *d*_0_ = 30 mm and length *l*_0_ = 30 mm, 60 mm, 90 mm, 120 mm and 150 mm are used. The material was selected due to its frequent use in the industry, especially in moderately loaded elements of machines and appliances, such as spindles, axles, rolls, plates, bolts, levers, wheel hubs etc. The chemical composition of the employed steel is shown in Table 3.

### 2.1. Experimental Tests

**Figure 2:**
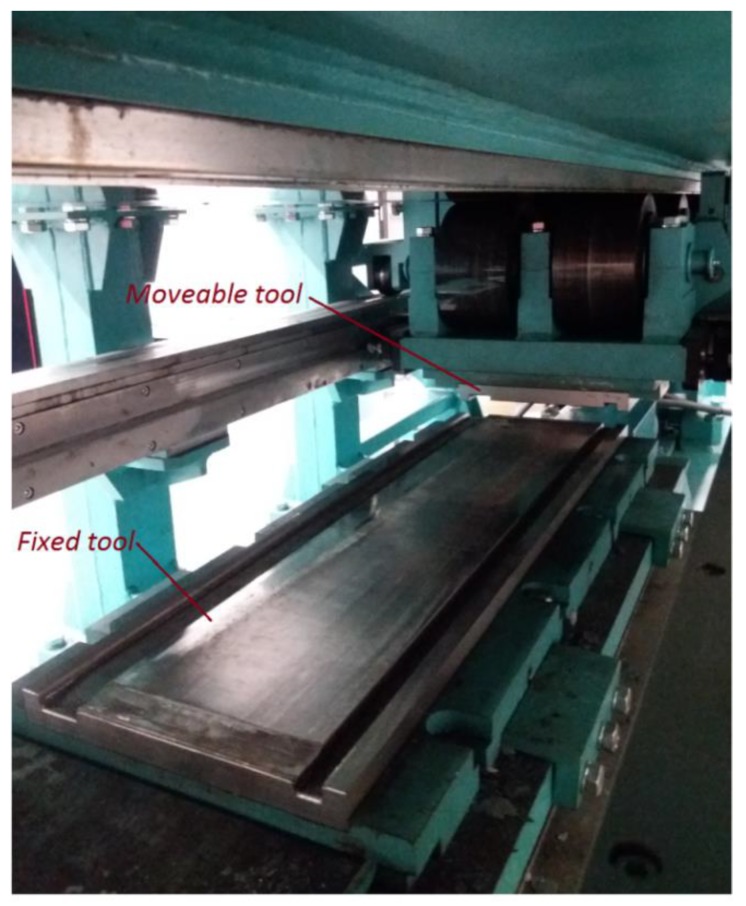
The working zone of a cross-rolling mill adapted to conduct the rotational compression of cylindrical samples.

The rotational compression tests were performed in a laboratory stand for cross-rolling, located at the Lublin University of Technology. The rolling mill (SIGMA SA, Barak, Lublin, Poland) used in the tests was equipped with a hydraulic drive and allows for performing rolling processes using up to 1000 mm long tools. The working zone of the rolling mill with the tools allowing one to perform the rotational compression tests is shown in Figure 2.

The samples were manufactured from a drawn bar with a Ø30 mm diameter, divided into pieces with the length equal 30 mm, 60 mm, 90 mm, 120 mm and 150 mm. Before forming, the samples were heated to the temperature *T* = 1150 °C in an electrical chamber furnace.

**Figure 3:**
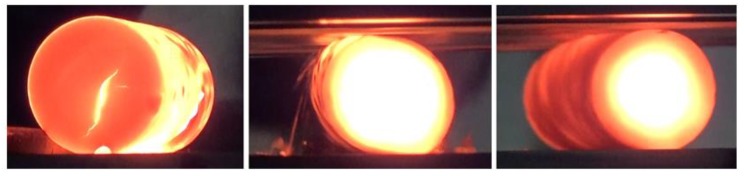
Rotational compression of a sample with the initial length equal 90 mm with the reduction ratio 1.15; the temperature of the billet 1150 °C.

An initial series of tests showed that the rotational compression process will be performed at the reduction ratio *δ* = 1.15, which was compliant with the distance between the working surfaces of tools 2*h* = 26.1 mm. It was the smallest possible distance at which the rotating motion occurred during the compression. It was also established that the length of the s path ought to be equal 315 mm. On this path, the compressed sample (Figure 3) rotated 3 ÷ 3.5 times and its front surfaces showed no signs of cracking.

**Figure 4:**
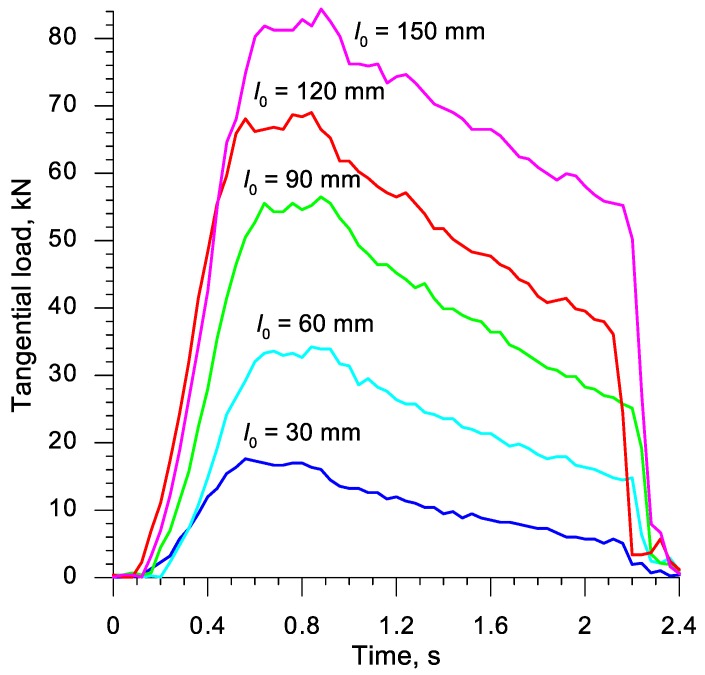
Distributions of the tangential force (moving the moveable tool) recorded during the rotational compression tests performer in laboratory conditions at the Lublin University of Technology.

In the actual series of rotational compression tests, three samples of each length were formed. During the tests, the tangential force required to move the upper tool was observed. The measured forces, the distribution of which was presented in Figure 4, were later used for the verification of the developed numerical model of the rotational compression test.

### 2.2. Numerical Analysis

The numerical simulation of the rotational compression process was conducted in the Simufact. Forming v.15 software, based on FEM. This software was used for analyses of cross- and skew-rolling processes multiple times [41,42,45,46,47,48,49], and the results of calculations remained compliant with the results of experimental testing.

**Figure 5:**
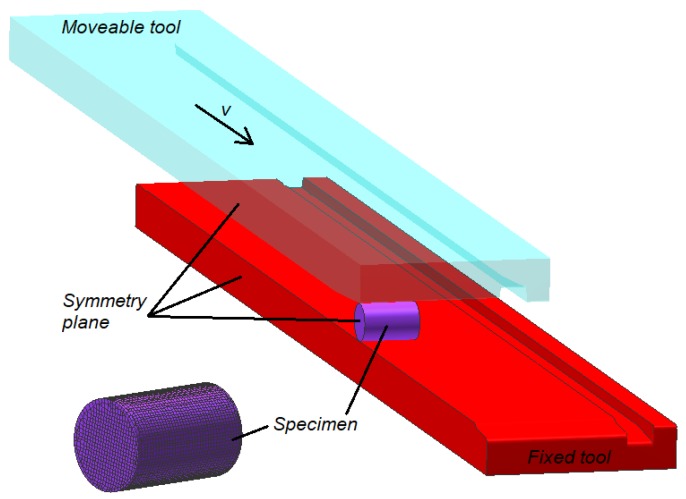
One of the geometrical models of the process of rotational compression used in the numerical analysis, in which the forming symmetry was applied.

The geometrical model of the researched test, created in the aforementioned FEM program, is shown in Figure 5. The model comprises of two flat tools, one of which (the upper tool) moves in-plane motion with the speed *v* = 300 mm/s and a billet modeled with the use of eight-noded elements. In order to simplify the calculations, it was assumed that the tools act as perfectly rigid bodies and compression symmetry were used. The shape and dimensions of the tools and the billet were similar to the ones used in experimental testing.

In the numerical analysis, a material model of C45 grade steel was used. In this model, the dependence of flow stress *σ_F_* on effective strain *ε*, strain ratio $\dot{\varepsilon }\;$and temperature *T* is rendered in the following form:

(9) $$\sigma_{F}=2859.8e^{-0.003125T}\varepsilon^{\left(0.00004466T-0.10126\right)}e^{\left(-0.00002725T+0.0008183\right)/\varepsilon }{\dot{\varepsilon }}^{\left(0.00015115T-0.002748\right)}.$$

The material model described with Equation (9) was downloaded from the data library of Simufact. Forming, the program used in the simulation. This model was successfully used multiple times by the authors in simulations of the rolling processes [50,51].

The friction was described using the Tresca model, where the friction factor was assumed to be *m* = 0.8. It was moreover stated that before the compression process the temperature of the sample is equal 1150 °C for its entire volume, whereas the temperature of the tools is 50 °C. The heat transfer coefficient between the material and the tools was assumed to equal 10,000 W/m^2^K.

**Figure 6:**
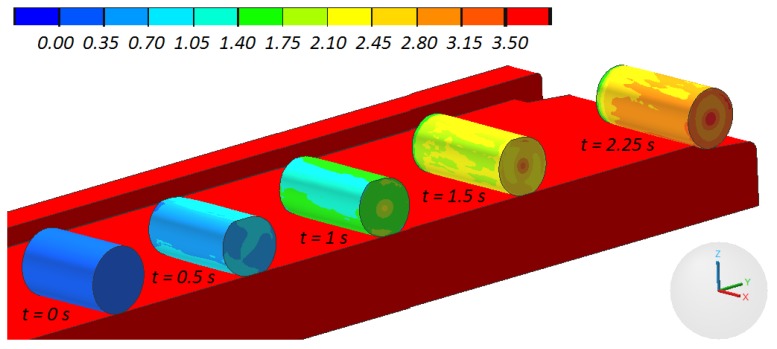
Progression of the sample shape, with the distribution of the effective strain, in the process of rotational compression of a sample with the initial length equal 90 mm (the upper tool was hidden for better visibility).

In total, five cases of compression were modeled, with the samples varying in length. An exemplary progression of the sample shape, prognosticated for one of the discussed cases of compression is shown in Figure 6. In all of the analyzed cases, the sample rolled relatively easily on the nether tool, as it was in the experimental tests.

**Figure 7:**
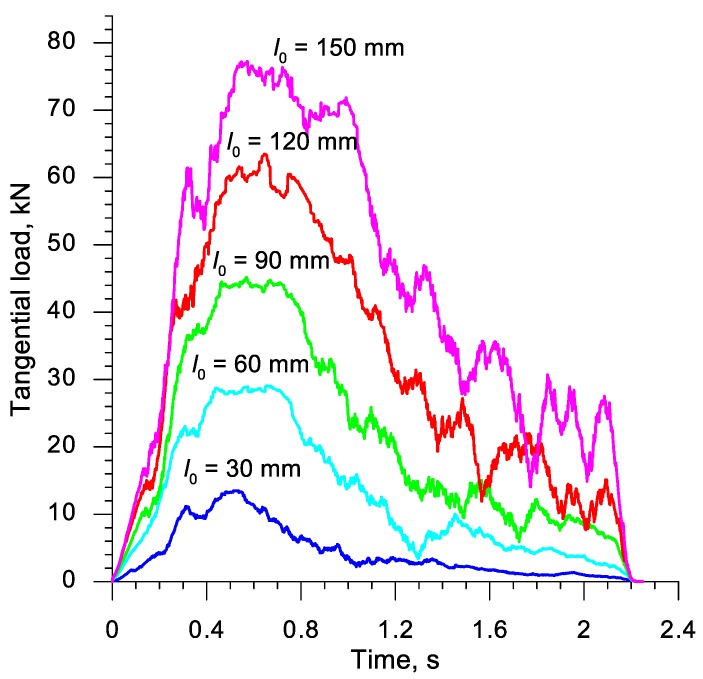
Distribution of the tangential force (moving the moveable tool) numerically prognosticated in Simufact. Forming v.15 software.

Figure 7 presents the distributions of the tangential force (moving the upper jaw), obtained in numerical simulations. A comparison of the distributions of forces shown in Figure 4 and Figure 7 indicates that the character of the processes is highly compliant. It was also observed that the forces from the calculations are more oscillatory, which is indubitably connected with the implicit model applied in the calculations, in which the convergence of the solution is searched iteratively. As far as the quantitative comparison of the forming forces is concerned, it is to be noted that the measured forces were higher than the ones obtained via calculations, with the difference of a few kN. This fact is caused by the additional resistance to the motion of the tool, connected to e.g., friction in guides, not included in the numerical analysis. It was ultimately stated that the developed FEM model of the rotational compression process renders the real test in a satisfactory manner.

## 3. Results and Discussions

**Figure 8:**
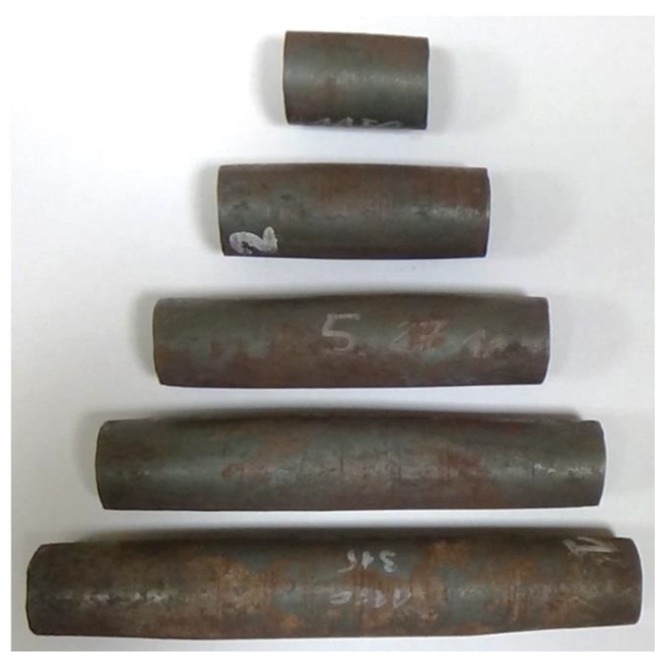
The samples deformed in the rotational compression of bars; respectively, from the top, the samples formed from billet with the length equal 30, 60, 90, 120 and 150 mm.

Rotational compression of cylindrical samples with the length *l*_0_ = 30 ÷ 150 mm, performed at the path *s* = 315 mm resulted in various levels of sample deformation. Figure 8 shows the samples deformed in experimental tests. An analysis of the shape of samples showed that only the shortest samples (*l*_0_ = 30 mm) retained their cylindrical shape. The shape of the remaining samples was barrel-like, with an oval outline of their cross-section in their midsection and circular outline at the end of the samples. The ovalization increased along with the length of the billet. Additionally, all samples had concave front surfaces, which indicates surface material flow during the rotational compression process.

**Figure 9:**
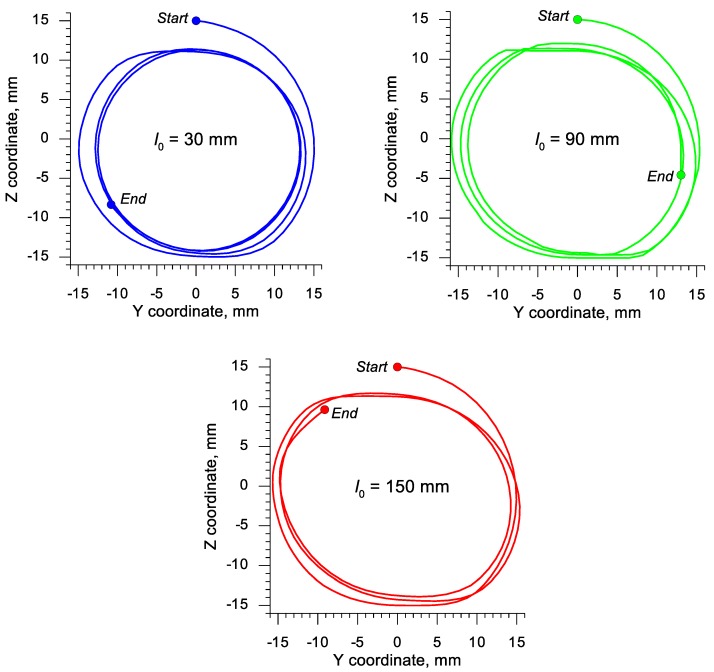
Trajectories of the displacement of the point located in the plane of symmetry and on the perimeter of the sample subjected to rotational compression, depending on the initial length of the sample *l*_0_.

In order to clarify the ovalization of samples, trajectories of motion for the point located in the plane of symmetry and on the outer surface of the sample were determined. According to the results of experimental tests, the most significant ovalization of the cross-section occurred in this section of the sample. The obtained trajectories of this point in three samples with the length 30, 90 and 150 mm are shown in Figure 9. An analysis of the data presented in this figure indicates that a circular outline in the plane of symmetry was obtained only for the shortest sample.

During the rotational compression of the sample with the initial length of 90 mm the ovalization was significantly decreased, but not eliminated. For the longest sample, it was only insignificantly decreased. This phenomenon can be easily explained. The reduction of ovalization is connected to elongating the sample, which is easier for shorter samples. Additionally, the oval shape of the cross-section makes it more difficult for the sample to be rolled, as a result of which the process is conducted with significant slippages. It is confirmed by the fact that during the rotational compression process at the same *s* path the shortest sample rotates over 3.5 times, the sample with *l*_0_ = 90 mm 3¼ times and the longest sample less than three times. It is, however, to be remembered that retaining the oval shape of the compression of the cross-section is necessary for the Mannesmann effect to occur.

**Figure 10:**
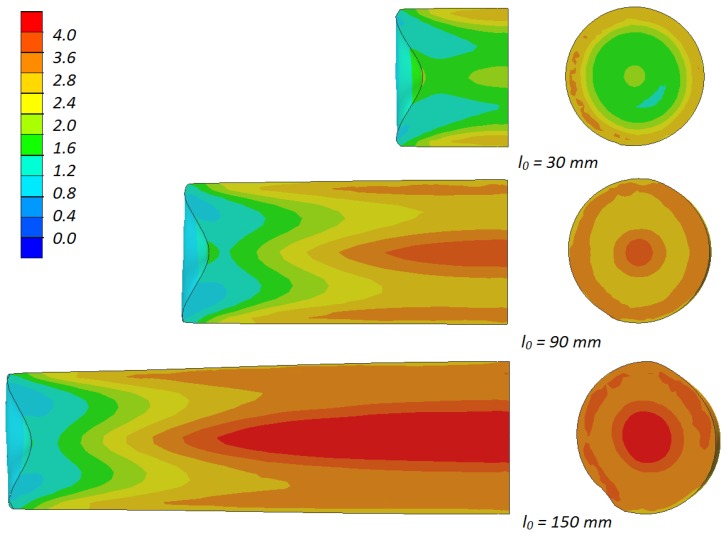
Distributions of effective strain in the samples (with the initial length *l*_0_) subjected to rotational compression in the path 315 mm; due to the symmetry of the process ½ samples were shown.

As a result of the rotational compression using flat tools, the material of the sample is intensively deformed. Its increase is shown in Figure 6. The very significant strain values marked in this figure occurring at an insignificant change of the sample dimensions indicate the predominant material flow in the tangential direction, causing high shear deformation to occur. The values of the strain in the sample depend mainly on its length, which was shown in Figure 10. The strain increases along with the length of the sample, with their highest values occurring in the axial area of the sample, which is indubitably connected to the ovalization of the cross-section of the sample, which causes the shear deformation to occur.

**Figure 11:**
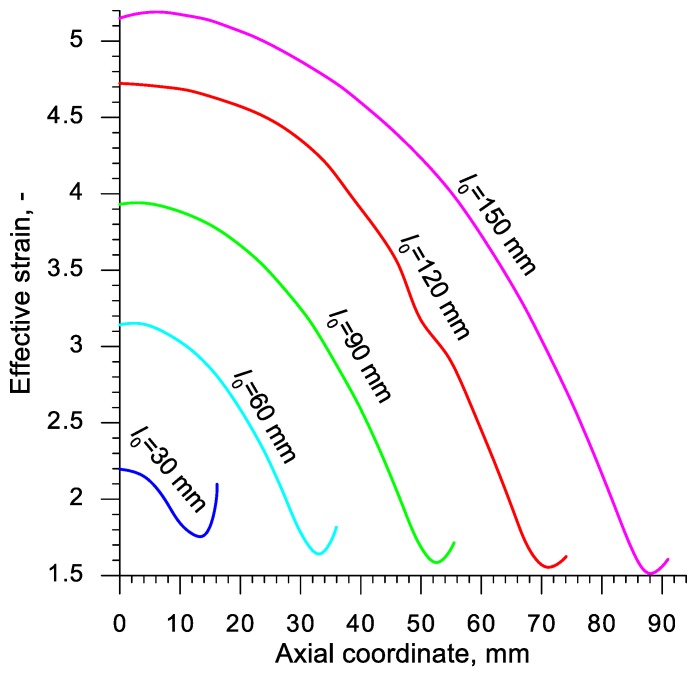
Numerically calculated distribution of effective strain in the axis of the sample, heated to 1150 °C and subjected to rotational compression on the path 315 mm; axial ordinate 0 describes the location of the plane of symmetry.

A quantitative comparison of the values of strain occurring in the axial area of the samples is presented in Figure 11. The distribution of strain was similar in all the analyzed cases. The highest values of this parameter can be observed in the middle of the sample and decrease progressively nearing the front surface. An insignificant local increase in strain occurring in the end of the sample is connected to the change of the stress state, which is more similar to torsion at this point. It was stated that the increase of the sample length from 30 to 150 mm caused the effective strain to increase c.a. 135%.

**Figure 12:**
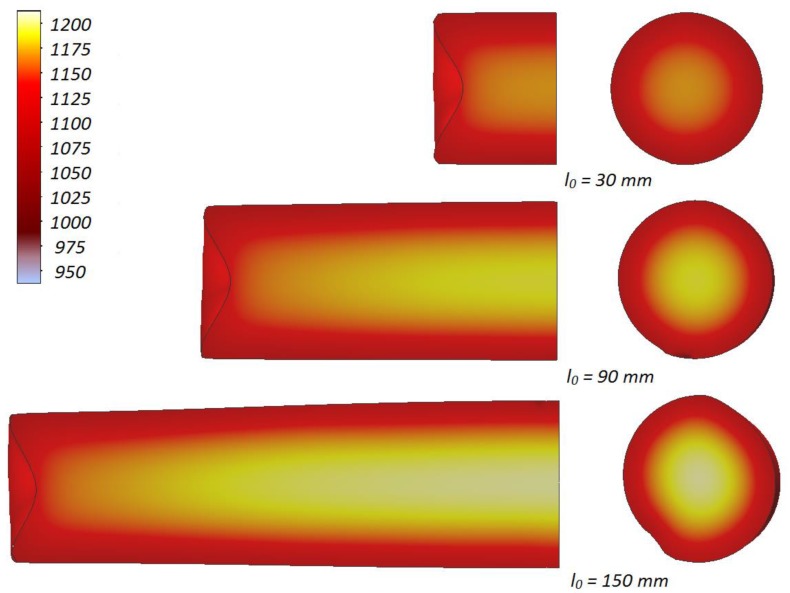
Distributions of temperature (w °C) in samples with the initial length *l*_0_, heated to *T* = 1150 °C and subjected to rotational compression in the path 315 mm; due to the symmetry of the process ½ samples was shown.

The intensive plastic deformation of the material significantly influences its temperature, which can be observed upon analyzing the data shown in Figure 12. As a result of the change of plastic work into heat, the temperature of the middle of the sample (in the axial area) not only does not decrease, but also increases despite the relatively long heating time. Simultaneously a decrease of temperature can be observed in the outer layers of the sample. This phenomenon is caused by the heat being transferred to significantly colder tools. This effect can be noticed also in Figure 3, depicting the process of one of the cases of rotational compression recorded during the tests performed in laboratory conditions.

**Figure 13:**
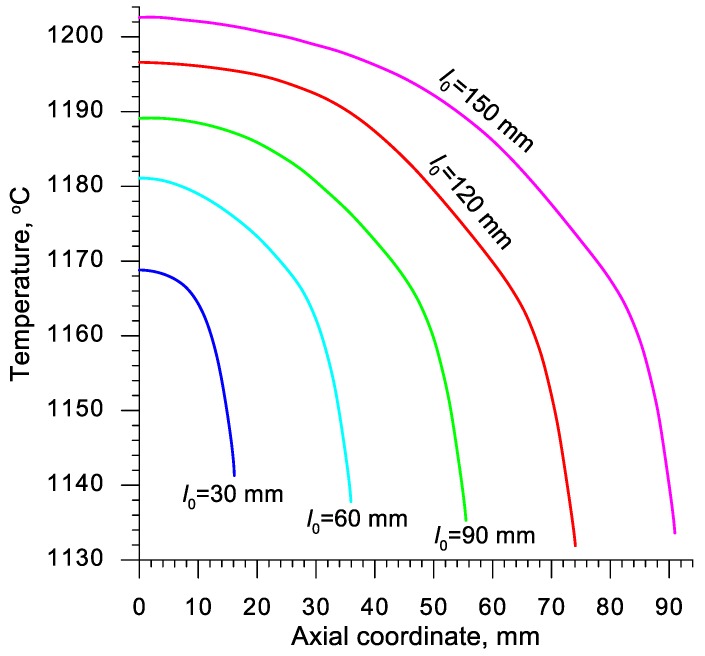
Numerically calculated distribution of temperature in the axis of the sample, heated to 1150 °C and subjected to rotational compression on the path 315 mm; axial ordinate 0 describes the location of the plane of symmetry.

Quantitative distribution of the temperature in the samples, numerically calculated, is shown in Figure 13. The data in this chart indicates that the highest temperature occurs in the middle of the sample and increases along with the length of the billet. In the case of the longest samples, the numerically prognosticated increase of temperature exceeds 50 °C, whereas for the shortest samples it is equal c.a. 19 °C. The temperature decreases gradually towards the ends of the sample, which is a result of the decrease in the heat generated as a result of a change of plastic work. The temperature of the material in the front surfaces of the samples is insignificantly lower than the temperature of the billet. It is, however, to be kept in mind that the numerical simulation does not include the transfer of the samples from the furnace and placing them on the nether tool. The time of those actions was c.a. 10 s and indubitably caused the temperature within the real process to decrease.

**Figure 14:**
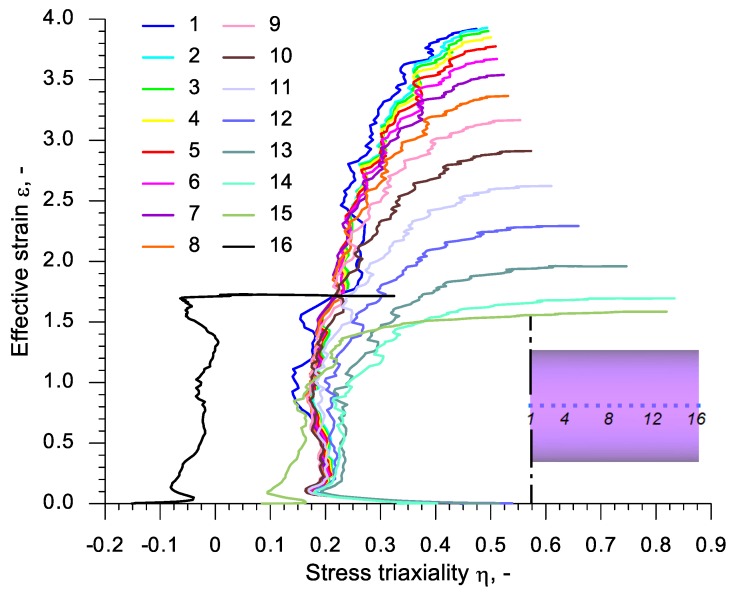
The dependency of effective strain on stress triaxiality in virtual sensors located in the axis of a sample with the length *l*_0_ = 90 mm, subjected to rotational compression.

**Figure 15:**
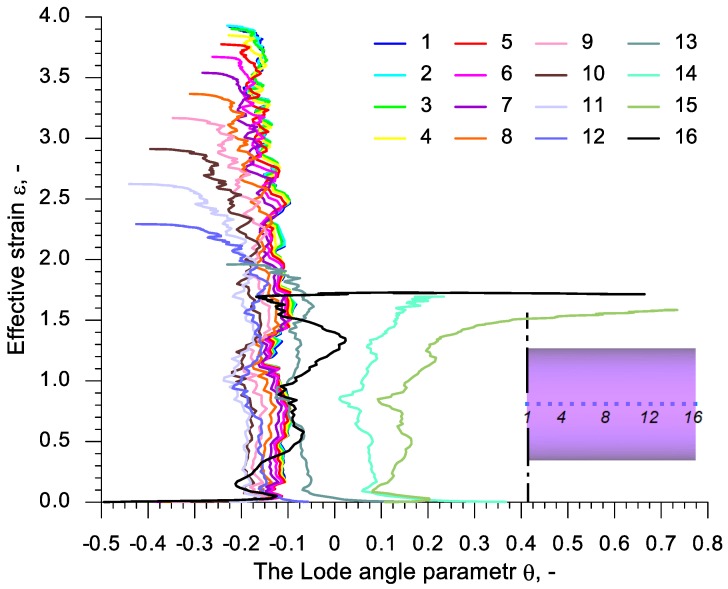
The dependency of effective strain on the Lode angle parameter in virtual sensors located in the axis of a sample with the length *l*_0_ = 90 mm, subjected to rotational compression.

In order to analyze the state of stress in the discussed rotational compression test, 16 virtual sensors were located at every 3 mm in the axis of the sample with the length *l*_0_ = 90 mm. The first sensor was located in the plane of symmetry and the 16th sensor in the front surface of the sample. These sensors monitored the values of the stress state parameters, such as stress triaxiality *η* and Lode angle parameter *θ*. The change to those parameters during the rotational compression is shown in Figure 14 and Figure 15. An analysis of the data presented in those figures indicates that excluding the end sensors (14, 15 and 16) the deformation of the material has a similar character. The maximum values of the increase of deformation occur at the stress triaxiality equal c.a. 0.2. In the final phase of the rotational compression, however, this parameter increases rapidly—the more so, the less significant the ovalization of the cross-section. Similar conclusions can be drawn on the subject of the distribution of the Lode angle parameter, which is in the range −0.2 ÷ −0.1 in the phase of a significant increase of deformation (excluding sensors 14, 15 and 16).

In order to compare the rotational compression of the cylindrical samples with the tests performed thus far it was necessary to determine the average values of both the stress triaxiality *η_av_* and Lode angle parameter *θ_av_*, calculated from the following dependencies:

(10) $$\eta_{av}=\frac{1}{\varepsilon }\int_{0}^{\varepsilon }\eta \;d\varepsilon ,$$

(11) $$\theta_{av}=\frac{1}{\varepsilon }\int_{0}^{\varepsilon }\theta \;d\varepsilon .$$

**Figure 16:**
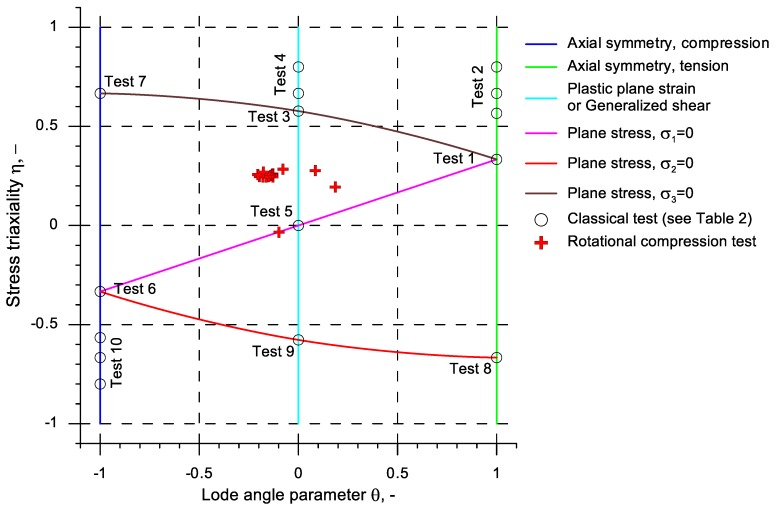
Representation of the state of stress on the surface *η* and *θ* with the tests used to calibrate the damage function.

The values determined for the 16 aforementioned virtual sensors were used to obtain the points on the plane of stress state that represents the state of stress in the new rotational compression test (Figure 16). Moreover, on this plane, the calibration tests proposed by Wierzbicki et al., presented in Table 2, were marked. An analysis of the data from this chart indicates that that the stress state in the test differs from the ones from the tests performed thus far. The points representing the state of stress in the middle of the rotational compressed sample are located in area defined by the ordinates *η* (0.2; 0.3) and *θ* (−0.2; −0.1). This location differed significantly from the points representing the state of stress at the end of the sample.

**Figure 17:**
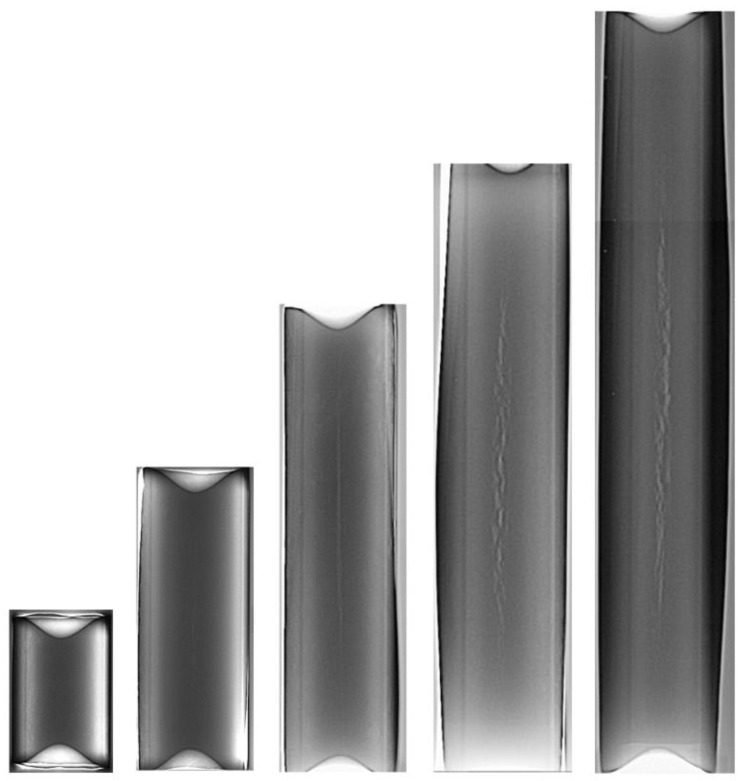
Exemplary roentgenograms of the rotationally compressed samples; dimensions of the samples—diameter 30 mm, initial length (from the left) 30, 60, 90, 120 and 150 mm.

The main objective of the rotational compression test of cylindrical samples was to determine the critical values of the damage function *C_i_*. Hence the samples after testing were subjected to non-destructive testing for internal cracking. In these tests, X-radiography was used. The obtained roentgenograms (Figure 17) were then analyzed for cracking, which showed that only the shortest samples (*l*_0_ = 30 mm) were cracking-free. In the remaining cases, the size of the detected cracks depended on the length of the samples. Upon averaging the results of the tests it was stated that the determined length of the cracking in relation to the length of the compressed sample is: 23.5% for the samples with *l*_0_ = 60 mm, 53.6% for the samples with *l*_0_ = 90 mm, 61.2% for the samples with *l*_0_ = 120 mm and 63.3% for the samples with *l*_0_ = 150 mm. It was also observed that in the case of the shorter samples the cross dimensions of the cracking were insignificant (c.a. a few tenths of a millimeter), whereas in the case of the longest samples the radial (cross) dimensions of the cracking increased to a few mm. Such propagation of the cracking was most likely caused by a different ovalization of the cross-section of the samples.

**Figure 18:**
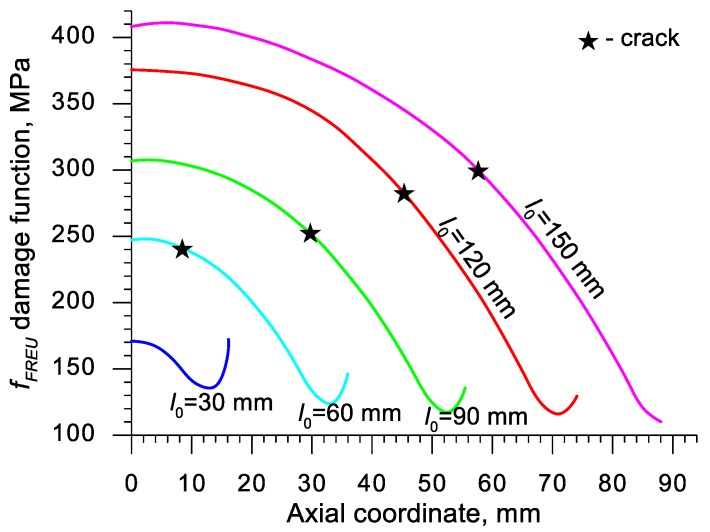
Distributions of the damage function in the axis of a rotationally compressed sample, determined with the use of the Freudenthal criterion.

**Figure 19:**
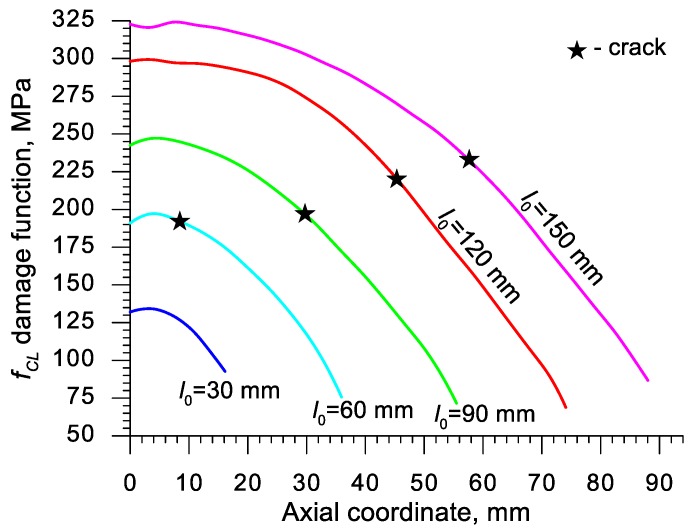
Distributions of the damage function in the axis of a rotationally compressed sample, determined on the basis of the Cockcroft and Latham criterion.

**Figure 20:**
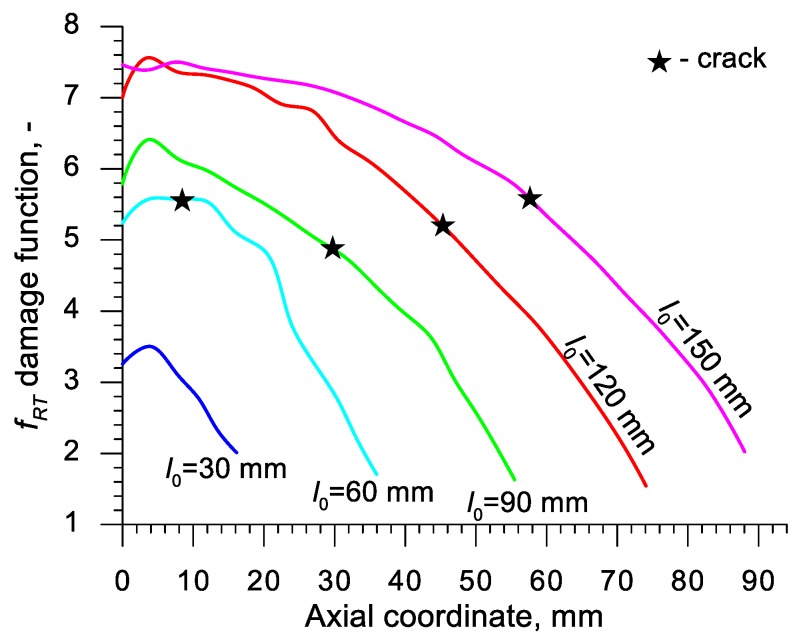
Distributions of the damage function in the axis of a rotationally compressed sample, determined on the basis of the Rice and Tracey criterion.

**Figure 21:**
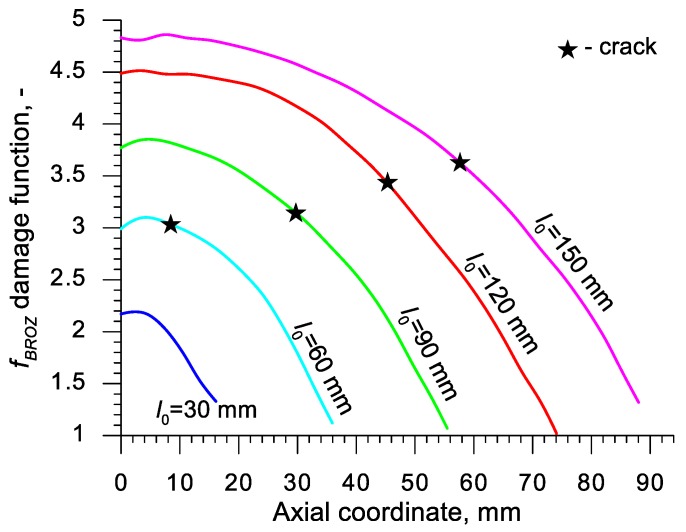
Distributions of the damage function in the axis of a rotationally compressed sample, determined on the basis of the Brozzo et al. criterion.

**Figure 22:**
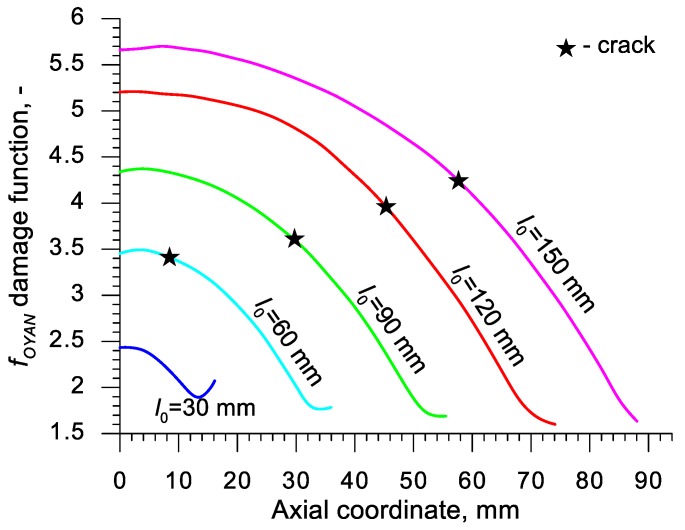
Distributions of the damage function in the axis of a rotationally compressed sample, determined on the basis of the Oyane criterion.

**Figure 23:**
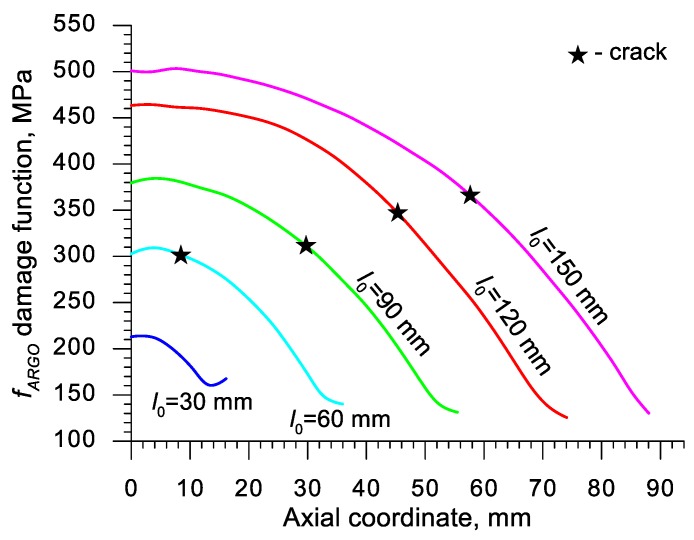
Distributions of the damage function in the axis of a rotationally compressed sample, determined on the basis of the Argon et al. criterion.

**Figure 24:**
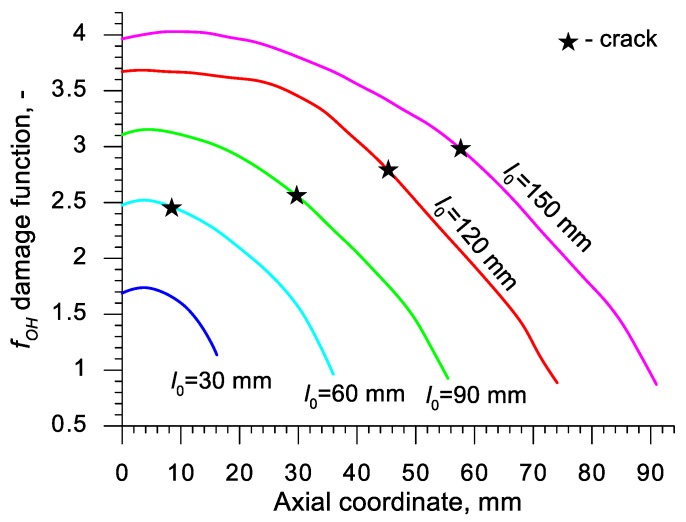
Distributions of the damage function in the axis of a rotationally compressed sample, determined on the basis of the Oh et al. criterion.

**Figure 25:**
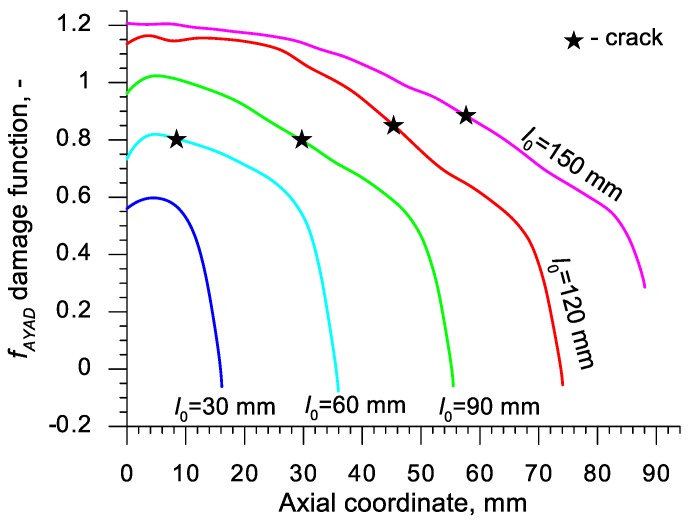
Distributions of the damage function in the axis of a rotationally compressed sample, determined on the basis of the Ayada criterion.

**Figure 26:**
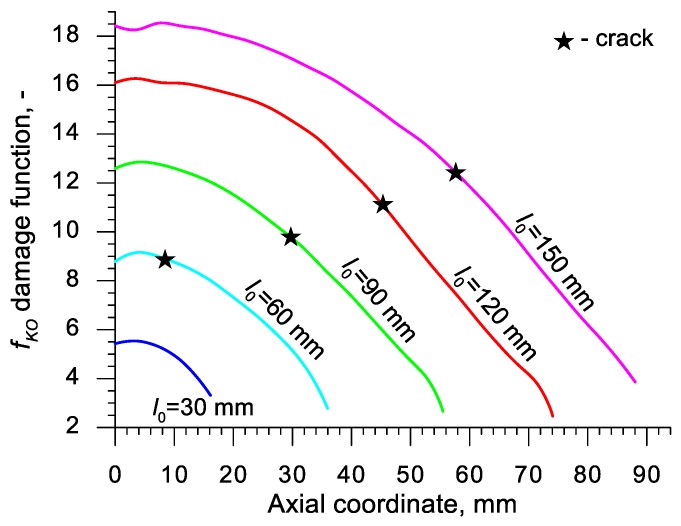
Distributions of the damage function in the axis of a rotationally compressed sample, determined on the basis of the Ko et al. criterion.

**Figure 27:**
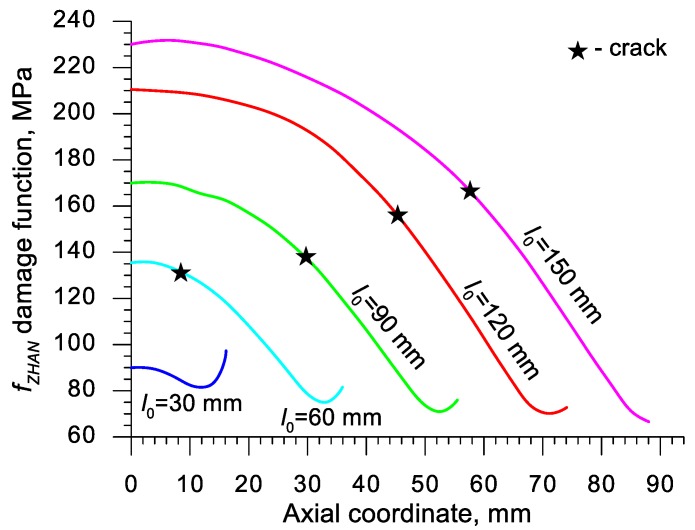
Distributions of the damage function in the axis of a rotationally compressed sample, determined on the basis of the Zhan et al. criterion.

Using the results of the numerical simulations the distributions of the damage function *f_i_* in the axis of the formed samples, presented in Figure 18, Figure 19, Figure 20,Figure 21, Figure 22, Figure 23, Figure 24, Figure 25, Figure 26 and Figure 27 were done. It was stated that all of the distributions of the damage function progressed in a similar manner. The maximum values are reached in the middle of the sample (axial ordinate 0 expresses the location of the plane of symmetry) and decrease in the direction to its front. Moreover, a strong dependence on the value of the damage function from the initial length of the sample was observed. In all of the discussed cases, the increase of the length *l*_0_ caused a very significant increase in the damage function. order to determine the critical value of the damage function, *C_i_* one ought to search the points (marked by ★) in which the cracking begins in the chart *f_i_*. The abscissae expressing the location of those points were determined based on the roentgenograms. The *f_i_* value in the point with the abscissa determined in such manner is equal to the searched critical value *C_i_*.

An analysis of the critical values of the damage function obtained in the above-presented manner shows a certain pattern, mainly an increase in both the value of *C_i_* and the initial length of the sample *l*_0_. This phenomenon may be caused by an increase of temperature in the axial area of the samples (see Figure 13), causing their deformability to rise and the cracking moment to delay. Alternatively, it could be a result of an increased propagation of the cracking in the radial (cross) direction, as a consequence of which the real material flow changes in relation to the one numerically prognosticated, where the material is constantly viewed as a continuous medium.

Considering the practical use of the critical values *C_i_* of the analyzed damage functions, their comparison was made (Table 4), where the lowest values of *C_i_* for each criterion are presented. It is advised to apply those values in an analysis of the cross- and skew-rolling processes of C45 grade steel products manufactured from billets in the temperature c.a. 1150 °C.

## 4. Conclusions

Based on the theoretical analyses and experimental testing performed, the following conclusions were drawn:

- A new method of calibrating the damage function was developed; in this method, the material cracks in the axial area of the sample as a result of the so-called Mannesmann effect;
- The developed calibrating test is recommended for the alloys of hot-worked metals, especially by cross- and skew rolling.
- The course of the rotational compression test (with the determined reduction ratio *δ*) depends strongly on the initial length of the sample; at *δ* = 1.15 the material cracks in the axial area of the sample when the length of the sample is at least two times greater than its diameter;
- In the rotational compression test, the material is the most deformed in the axial area of the sample; those deformations are caused by the intensive material flow in the tangential direction;
- Despite the relatively long duration of the rotational compression test the material temperature not only does not decrease, but also increases; the rise of temperature is a result of the change of plastic work into heat;
- The state of stress occurring in the axial area of the samples subjected to rotational compression differs significantly from the stress occurring in the calibrating tests applied thus far; during the most significant increase of effective strain this state is expressed by the averaged value of stress triaxiality *η_av_* = (0.2; 0.3) and the averaged value of the Lode angle parameter *θ_a_*_v_ = (0.2; −0.1);
- For a practical application, it is advised to use the critical values of the damage function of C45 grade steel presented in Table 4; the aforementioned values were determined for ten damage criteria and are vital for hot forming in the temperature c.a. 1150 °C.

## Figures and Tables

**Table 1:** Selected criteria of ductile fracture used for the presented analysis [21,22,23,24,25,26,27,28,29,30].

Abbreviation	Criterion (Year)	Formula
FREU	Freudenthal (1950)	$f_{FREU}=\int_{0}^{\varepsilon }\sigma_{i}d\varepsilon$
CL	Cockroft and Latham (1968)	$f_{CL}=\int_{0}^{\varepsilon }\sigma_{1}d\varepsilon$
RT	Rice and Tracey (1969)	$f_{RT}=\int_{0}^{\varepsilon }\exp\left(\frac{3}{2}\eta \right)d\varepsilon$
BROZ	Brozzo et al. (1972)	$f_{BROZ}=\int_{0}^{\varepsilon }\frac{2\sigma_{1}}{3\left(\sigma_{i}-\sigma_{m}\right)}d\varepsilon$
OYAN	Oyane (1972)	$f_{OYAN}=\int_{0}^{\varepsilon }\left(1+A\eta \right)d\varepsilon$
ARGO	Argon et al. (1975)	$f_{ARGO}=\int_{0}^{\varepsilon }\left(\sigma_{m}+\sigma_{i}\right)d\varepsilon$
OH	Oh et al. (1979)	$f_{OH}=\int_{0}^{\varepsilon }\frac{\sigma_{1}}{\sigma_{i}}d\varepsilon$
AYAD	Ayada (1984)	$f_{AYAD}=\int_{0}^{\varepsilon }\eta d\varepsilon$
KO	Ko et al. (2007)	$f_{KO}=\int_{0}^{\varepsilon }\frac{\sigma_{1}}{\sigma_{i}}\left(\langle 1+3\eta \rangle \right)d\varepsilon$
ZHAN	Zhan et al. (2009)	$f_{ZHAN}=\int_{0}^{\varepsilon }\left(\sigma_{i}-\sigma_{m}\right)d\varepsilon$

Where: *A*—Material constant. Further on in the study, it is assumed that *A* = 0.424 [40].

**Table 2:** A presentation of the classic tests used for calibrating the damage function, developed by Wierzbicki et al. [17,34].

Test No.	Characterization	Stress Triaxiality *η*	Lode Angle Parameter *θ*
1	Smooth bars, tension	$\frac{1}{3}$	1
2	Notched bars, tension	$\frac{1}{3}+\sqrt{2}\ln\left(1+\frac{a}{2R}\right)$	1
3	Plastic plane strain, tension	$\frac{\sqrt{3}}{3}$	0
4	Flat grooved plates, tension	$\frac{\sqrt{3}}{3}\left[1+2\ln\left(1+\frac{t}{4R}\right)\right]$	0
5	Torsion or shear	0	0
6	Cylinders, compression	$-\frac{1}{3}$	−1
7	Equi-biaxial plane stress tension	$\frac{2}{3}$	−1
8	Equi-biaxial plane stress compression	$-\frac{2}{3}$	1
9	Plastic plane strain, compression	$-\frac{\sqrt{3}}{3}$	0
10	Notched bars, compression	$-\left[\frac{1}{3}+\sqrt{2}\ln\left(1+\frac{a}{2R}\right)\right]$	−1

Where: *R*—Radius of the notch or groove, *a*—Radius of the bar in the notch area, *t*—Thickness of the grooved sample with a flat groove.

**Table 3:** Chemical composition of C45 grade steel (% by weight).

C	Mn	Si	P	S	Cr	Ni	Mo	Cu	Fe
0.42–0.5	0.5–0.8	0.1–0.4	max 0.04	max 0.04	max 0.03	max 0.3	max 0.1	max 0.3	balance

**Table 4:** Critical values of the damage function determined for C45 grade steel, formed at 1150 °C.

*C_FREU_*	*C_CL_*	*C_RT_*	*C_BROZ_*	*C_OYAN_*	*C_ARGO_*	*C_OH_*	*C_AYAD_*	*C_KO_*	*C_ZHAN_*
241.2	192.4	4.88	3.04	3.42	301.9	2.46	0.81	8.90	131.6
